# Predicting 6-month mortality of patients from their medical history: Comparison of multimorbidity index to Deyo–Charlson index

**DOI:** 10.1097/MD.0000000000032687

**Published:** 2023-02-03

**Authors:** Farrokh Alemi, Sanja Avramovic, Mark Schwartz

**Affiliations:** a Department of Health Administration and Policy, George Mason University, Fairfax, VA; b Department of Population Health, NYU Grossman School of Medicine, NY.

**Keywords:** diabetes, hospice admission, prognosis, severity of illness

## Abstract

While every disease could affect a patient’s prognosis, published studies continue to use indices that include a selective list of diseases to predict prognosis, which may limit its accuracy. This paper compares 6-month mortality predicted by a multimorbidity index (MMI) that relies on all diagnoses to the Deyo version of the Charlson index (DCI), a popular index that utilizes a selective set of diagnoses. In this retrospective cohort study, we used data from the Veterans Administration Diabetes Risk national cohort that included 6,082,018 diabetes-free veterans receiving primary care from January 1, 2008 to December 31, 2016. For the MMI, 7805 diagnoses were assigned into 19 body systems, using the likelihood that the disease will increase risk of mortality. The DCI used 17 categories of diseases, classified by clinicians as severe diseases. In predicting 6-month mortality, the cross-validated area under the receiver operating curve for the MMI was 0.828 (95% confidence interval of 0.826–0.829) and for the DCI was 0.749 (95% confidence interval of 0.748–0.750). Using all available diagnoses (MMI) led to a large improvement in accuracy of predicting prognosis of patients than using a selected list of diagnosis (DCI).

## 1. Introduction

Many studies use the Charlson index and its improved variants (e.g., ^[1–5]^). As of publication of this article, there were 37,619 articles referring to these indices. The Charlson index, and its variants, rely on selected illnesses and ignore all other diseases, including obvious predictors of mortality such as coma. In this report, we compare performance of a multimorbidity index (MMI), an index that relies on all patients’ diagnoses to the Deyo version of the Charlson index (DCI), a leading example of indices that rely on a selective list of diseases to predict 6-month mortality.

Several studies have shown that indices that rely on all diseases lead to more accurate predictions. The MMI index has been shown to be more accurate than either the Charlson, the Deyo, or the Elixhauser indices.^[6]^ The M3 index (also an index that relies on multiple morbidities), was shown to be more accurate than Charlson and Elixhauser indices.^[7,8]^ Despite these findings, the use of Charlson index and its variants continues. Part of the reason could be that MMI indices, while more comprehensive, are less clinically intuitive; in the sense that clinicians cannot track thousands of different diseases without relying on a decision aid. Another reason could be that clinicians may not consider the improvement in accuracy as large enough to be clinically meaningful.^[9]^ In this paper, we modified the MMI to make it intuitively more meaningful to clinicians, while maintaining its comprehensive reliance on all diagnoses. In particular, we organized diagnoses within body system and examined progression of disease within body system.

## 2. Methods

### 2.1. Ethics review

This study was approved by the New York University, George Mason University and Veterans Administration’s Institutional Review Boards.

### 2.2. Study design

We conducted a retrospective cohort study.

### 2.3. Setting

This study examined data from the “United States Veterans Administration Diabetes Risk” national cohort.^[10]^

### 2.4. Participants

This cohort included 6,082,018 non-diabetic veterans receiving primary care at the Veteran Administration health care system, from January 1, 2008 to December 31, 2018. Patients had to have at least 2 primary care visits at least 30 days apart. Patients who had no encounters for 2 years were censored as lost to follow-up.

### 2.5. Dependent variable

The outcome of interest was 6-month mortality. Previous publications have typically used 1-year mortality rates. There is no reason to expect that the relative performance of these 2 indices depends on 6-month or 12-month outcomes. A disease that predicts mortality risk in 6 months is also often predictive of mortality risk in 12 months. We chose to report a 6-month mortality risk because it more closely relates to the use of hospice under Medicare rules; and shows how a clinical decision might be affected by these 2 indices.

### 2.6. Independent variables

There were 7805 unique diagnoses in the Veterans Administration Diabetes Risk cohort. These diagnoses were identified through International Classification of Diseases (ICD) at hospital discharge, or through outpatient visits. Since the majority of our data comes from years in which ICD version 9 was used to code discharge diagnosis, codes for years in which version 10 was used were converted to version 9.

Historically, MMI indices have used all the diagnoses without classifying them into broad categories. To help clinicians get a better understanding of changes in the patients’ conditions, in this study we classify 7805 diagnoses codes into 19 body-systems. The 19 body systems are indicated by the first 3 digits of the ICD code, including: infectious and parasitic diseases; neoplasms; endocrine, nutritional and metabolic diseases, and immunity disorders; diseases of the blood and blood-forming organs; mental disorders; diseases of the nervous system and sense organs; diseases of the circulatory system; diseases of the respiratory system; diseases of the digestive system; diseases of the genitourinary system; complications of pregnancy, childbirth, and the puerperium; diseases of the skin and subcutaneous tissue; diseases of the musculoskeletal system and connective tissue; congenital anomalies; conditions originating in the perinatal period; ill-defined conditions; injury and poisoning; external causes of injury, E codes; and social determinants of illness using V codes. Within each body system, we selected the worst disease of the patient, for example the disease with highest likelihood ratio (LR) of mortality within 6 months. was calculated for each disease. This method of variable constructions allows one to map the medical history of a patient into the body systems and select the worst diagnosis (most predictive of mortality) within each body system.

The Deyo–Charlson index (DCI) also started with the same 7805 unique diagnoses in our cohort but classified these diagnoses into 17 conditions^[11,12]^: myocardial infarction, congestive heart failure, peripheral vascular disease, cerebrovascular disease, dementia, chronic pulmonary disease, rheumatic disease, peptic ulcer disease, mild liver disease, diabetes without chronic complications, diabetes with chronic complications, hemiplegia or paraplegia, renal disease, any malignancy, moderate or severe liver disease, metastatic solid tumor, and acquired immunodeficiency syndrome/human immunodeficiency virus. In addition, the DCI included the age of the patient.

### 2.7. Measurement of LRs

For the MMI, the odds of mortality within each body system was assessed in 2 steps. First, for each disease the LR of mortality in the next 6 months was calculated using the training data set. A LR is calculated by dividing the prevalence of the disease among patients who have died in 6 months by the prevalence of the same disease among patients who have not died:


$$\begin{array}{l} LR=\frac{p(Disease\;\;|\;\;Dead\;in\;6\;months)}{p(Disease\;\;|\;\;Not\;Dead\;in\;6\;months)} \end{array}$$

For diseases in which all patients die, or all patients live, the LR cannot be calculated from above formula. When all patients die, the LR is approximated as the number of patients with the disease plus 1, that is, LR=n+1;  where n is the number of patients in the training sample with the disease. For diseases in which all patients survive, the LR is calculated as 1 over the sum of number of patients with the disease plus 1, that is, LR=1/(n+1).

In addition to the above adjustments, for rare diseases that occurred <100 times in the training data set, the sample size was too small for an accurate estimate of the LR. For these rare diseases, the LR for the diagnosis was replaced with the LR of the broader concept of the disease, using the ICD ontology to define the broader concept.^[13]^ For example, there were only 69 cases of “malignant neoplasm of other specified sites of gallbladder and extrahepatic bile ducts (ICD code 156.8)”; but there were 2004 cases of the broader disease concept that included malignant neoplasms of gallbladder, extrahepatic bile ducts, and ampulla of vater combined (i.e., ICD codes 156.0, 156.1, 156.2, 156.8, and 156.9).

### 2.8. Definition of the worst disease in each body system

The ICD assigns diseases to different body systems. Diseases within the same body system were ranked in order of the LRs for 6-month mortality associated with the disease. The worst disease within the body system was the one with the highest LR for mortality. All other diseases in the body system were ignored and only the LR associated with the worst disease was used in calculating overall prognosis of each patient.

Note that body systems include catch-all categories such as “Ill defined” system or “Social determinants of illness.” These categories lead to surprising distinctions among diseases. For example, malignant ascites is classified under “ill defined” category; while malignant pleural effusion is in the pulmonary body system.

### 2.9. Prognosis of each patient

The predicted odds of mortality was calculated as the product of the LRs associated with the worst disease in the body system. If b indicates the body system, then odds of mortality for a patient was calculated as:


$$\begin{array}{l} Odds\;of\;Mortality=\underset{b}{\prod }\;LR\;of\;Worst\;Disease_{b} \end{array}$$

### 2.10. Measurement of accuracy

Because the MMI relied on a large number of predictors, 90% of the data were used for training and 10% for validation. The accuracy of predictions was calculated using area under the receiver operating curve (ROC), where 0.5 indicates a random prediction and 1.0 indicates a perfect prediction.

### 2.11. Sources of bias

No effort was made to statistically control for confounding among the comorbidities and diseases. No effort was made to examine subgroups of patients or interactions among the comorbidities.

### 2.12. Missing information

If the date of mortality was absent, the patient was assumed to be alive. If a diagnosis was not recorded in the medical record, we assumed the patient did not have the diagnosis.

## 3. Results

A detailed description of the cohort is available elsewhere.^[10]^ There were 6,082,246 unique patients in the cohort. The average age of patients was 58.3 years, 91.7% were male, 72.5% were White, 15.1% were Black, 2.8% were Asian, American Indian, or Native Hawaiian, and 5.4% were of Hispanic ethnicity. The average follow-up was 5.8 years. Among the 6.082 million patients, 936,596 (17.7%) died during the study period.

The MMI index is built on progression of illness within body systems. Table 1 shows an example of data that can describe this progression. This Table has been simplified to make it easier to present. A more complete Table is available through the online Appendix S1, Supplemental Digital Content, http://links.lww.com/MD/I348. The rows in the Table show body systems. Within each row, the columns mark risks of mortality associated with different diseases. For ease of display, we arbitrarily present the data in 7 risk categories only. In MMI, risk is measured as a continuous variable. In addition, for ease of reporting, we are presenting only 1 disease per cell in the Table. Progression of risk means a sequence of events over time with increasing risk of mortality. Thus, if a patient moves from the left-most column in the Table to the right, then we are seeing a worsening situation, a progression of illness.

**Table 1:** Examples of diseases marking risk within body systems.

System	Very low (<.22)	Low (.22 to.33)	Reduced (.33–.66)	No risk (.66–1.5)	Elevated (1.5–3)	High (3–6)	Very high (>6)
Blood system	Other specified diseases of blood (LR = 0, ICD = 289.8),	Methemoglobinemia (LR = .31, ICD = 289.7)	Other specified aplastic anemias (LR = .65, ICD = 284.89)	Anemia in neoplastic disease (LR = 1.14, ICD = 285.22)	Hemophagocytic syndromes (LR = 1.93, ICD = 288.4)		
Circulatory system	Iatrogenic hypotension (LR = 0, ICD = 458.2)	Esophageal varices with bleeding (LR = .32, ICD = 456.0)	Thoracoabdominal aneurysm ruptured (LR = .65, ICD = 441.6)	Other acute rheumatic heart disease (LR = 1, ICD = 391.8)	Cardiac arrest (LR = 2.26, ICD = 427.5)		
Digestive system	Other suppurative peritonitis (LR = 0, ICD = 567.2)	Perforation of intestine (LR = .33, ICD = 569.83)	Hepatic coma (LR = .65, ICD = 572.2)	Vomiting of fecal matter (LR = 1.21, ICD = 569.87)		Hepatorenal syndrome (LR = 3.02, ICD = 572.4)	
Endocrine system	Volume depletion disorder (LR = 0, ICD = 276.5)	Glucocorticoid deficiency (LR = .32, ICD = 255.41)	Other amyloidosis (LR = .6, ICD = 277.39)	Other severe protein-calorie malnutrition (LR = .8, ICD = 262.)	Tumor lysis syndrome (LR = 1.58, ICD = 277.88)		
Ill-defined	Asphyxia (LR = 0, ICD = 799.0)	Hemorrhage from throat (LR = .32, ICD = 784.8)	Other shock without mention of trauma (LR = .65, ICD = 785.59)	Septic shock (LR = 1.48, ICD = 785.52)	Coma (LR = 2.48, ICD = 780.01)		Malignant ascites (LR = 7.22, ICD = 789.51)
Infectious system	Sporotrichosis (LR = .22, ICD = 117.1)	Opportunistic mycoses (LR = .32, ICD = 118.)	Methicillin resistant *Staphylococcus aureus septicaemia* (LR = .65, ICD = 038.12)	*Candidal endocarditis* (LR = 1.02, ICD = 112.81)		Other and unspecified Creutzfeldt–Jakob disease (LR = 3.04, ICD = 046.19)	
Injury diagnoses	Late effect of injury due to war operations (LR = 0, ICD = E999.)	Other infusion reaction (LR = .32, ICD = 999.88)	Closed fracture of unspecified vertebra with spinal cord injury (LR = .65, ICD = 806.8)	Severe sepsis (LR = 1.04, ICD = 995.92)			
Mental health disorders	Other specific developmental learning difficulties (LR = 0, ICD = 315.2)	Delirium due to conditions classified elsewhere (LR = .31, ICD = 293.0)	Senile dementia with delirium (LR = .57, ICD = 290.3)				
Musculoskeletal system	Acquired deformity of nose (LR = 0, ICD = 738.0)	Chronic osteomyelitis involving multiple sites (LR = .32, ICD = 730.19)	Pathologic fracture unspecified site (LR = .61, ICD = 733.10)	Pathologic fracture of the humerus (LR = 1.49, ICD = 733.11)			
Neoplasm	Neoplasm of uncertain behavior of other lymphatic and hematopoietic tissues (LR = 0, ICD = 238.7)	Acute lymphoid leukemia w/o mention of remission (LR = .33, ICD = 204.00)	Merkel cell carcinoma of unspecified upper limb (LR = .66, ICD = 209.33)	Kaposi sarcoma gastrointestinal sites (LR = 1.5, ICD = 176.3)	Secondary neuroendocrine tumor of bone (LR = 2.89, ICD = 209.73)	Secondary malignant neoplasm of brain and spinal cord (LR = 3.58, ICD = 198.3)	
Nervous system	Mixed conductive and sensorineural hearing loss (LR = 0, ICD = 389.2)	Other encephalopathy (LR = .33, ICD = 348.39)	Metabolic encephalopathy (LR = .65, ICD = 348.31)	Myelopathy in other diseases classified elsewhere (LR = 1.46, ICD = 336.3)	Neoplasm related pain (acute) (chronic) (LR = 2.9, ICD = 338.3)		Brain death (LR = 46, ICD = 348.82)
Other factors	Special screening examination other specified viral diseases (LR = 0, ICD = V73.89)	Attention to gastrostomy (LR = .32, ICD = V55.1)	Personal history of antineoplastic chemotherapy (LR = .63, ICD = V87.41)	Do not resuscitate status (LR = .98, ICD = V49.86)		Encounter for palliative care (LR = 5.83, ICD = V66.7)	
Respiratory system	Other diseases of trachea and bronchus (LR = 0, ICD = 519.1)	Pneumonia due to *S aureus* (LR = .33, ICD = 482.41)	Null (LR = .63, ICD = 512.82)	Acute interstitial pneumonitis (LR = 1.1, ICD = 516.33)		Malignant pleural effusion (LR = 3.28, ICD = 511.81)	

LR refers to likelihood ratio within 6 months. Risk was measured as LR. ICD refers to 7508 International Classification of Disease codes within our data. LRs were calculated in 100+ cases with the disease.

An example can further demonstrate the progression of illness within a body system. Within the circulatory body system, we have listed 5 example diseases. These listed in order of worsening LRs of 6-month mortality are: iatrogenic hypotension, esophageal varices with bleeding, thoracoabdominal aneurysm ruptured, acute rheumatic heart disease, and cardiac arrest. Each of these diseases have a particular likelihood of mortality. Moving from hypotension to cardiac arrest marks progression of illness in the circulatory body system, if it occurs in an individual patient.

For combination of diseases within the same body system, the MMI scores the risk associated with the worst disease within each body system. Thus, for a patient with both iatrogenic hypotension and cardiac arrest, the MMI ignores the hypotension and scores only the cardiac arrest.

**Figure 1.**
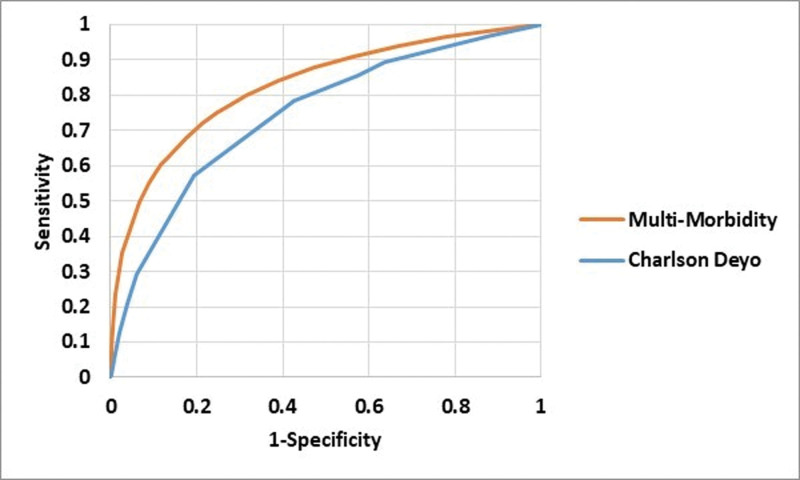
Accuracy of multimorbidity and Deyo variant of Charlson (DC) index.

Figure 1 shows the sensitivity and specificity of the 2 indices in predicting mortality, over the range of scores for each. The area under the ROC for the MMI was 0.828 (95% confidence interval of 0.826–0.829). The area under the ROC for the DCI was 0.749 (95% confidence interval of 0.748–0.750). The difference between MMI and DCI accuracy was statistically significant at α levels of 0.05. The relative improvement in accuracy was 10.7%.

## 4. Discussion

MMI was significantly (*α* = 0.05) more accurate than DCI by 0.08 points in the area under the curve (AUC). This level of improvement in AUC corresponds 10.7% relative increase in accuracy. A 2% improvement in the AUC, is considered to be clinically meaningful increase.^[14]^ Therefore, the 8% absolute improvement is large enough to not only be statistically significant but also clinically meaningful.

There are many reasons why MMI is more accurate than DCI. One reason for this improvement in accuracy is because rare diseases, for example, coma, are often predictive of poor prognosis. Comprehensive indices such as MMI include all rare diseases. Selective indices, such as DCI, do not; and perhaps herein lies why the 2 indices perform differently. If we consider disease that occur in <100 patients as rare, nearly 50% of diseases were rare in our sample. Ignoring any rare disease may not matter; but ignoring all of them will cumulatively lead to ignoring half of the data – not an infrequent event. It could lead to large drop in accuracy.

The difference in performance of the 2 indices cannot be due to severity of illness within the diagnoses. The ICD, for the most part, ignores severity of illness within each diagnosis. Exceptions exist. For example, diabetes and diabetes with complications have separate codes, but for the most part this classification system ignores severity within the diagnoses. Both MMI and DCI rely on the same set of diagnoses and thus both ignore severity of illness within the diagnoses. Therefore, the differential performance of these 2 indices cannot be due to severity within diagnoses.

It is possible that the differential performance of these 2 indices is an artifact of how we organized the study. DCI parameters was constructed on a different database and validated in our data. In contrast, the parameters of the MMI were derived from the 90% training set and then cross-validated on 10% of the data, both coming from the same database. When an index is developed on randomly set-aside data, it may be more accurate than when validated on entirely different database.

We are cognizant that the differential performance of these 2 indices was shown only in 1 database that focused on experiences of veterans. Veterans differ from the general population in distinct ways, including the fact that veterans report more comorbidities than the general population.^[15]^ It is possible that our findings cannot be generalized to non-veteran populations.

If MMI is more accurate than DCI, it is important that clinicians and investigators start using the more accurate index because when prognostic indices are in error, the treatment recommended to the patient may change. For example, referral of patients to hospice may change if prognostic indices are in error. In addition, when prognostic indices are in error, conclusions of policy analysis may change. A policy that saves lives may erroneously be undervalued as causing excess mortality. Using more accurate prognostication could lead to more appropriate decisions for individual patients and population of patients.

## Author contributions

**Conceptualization:** Farrokh Alemi.

**Data curation:** Mark Schwartz.

**Formal analysis:** Sanja Avramovic.

**Funding acquisition:** Mark Schwartz.

**Investigation:** Farrokh Alemi, Mark Schwartz.

**Methodology:** Farrokh Alemi.

**Project administration:** Sanja Avramovic, Mark Schwartz.

**Supervision:** Farrokh Alemi, Mark Schwartz.

**Writing – original draft:** Farrokh Alemi.

**Writing – review & editing:** Farrokh Alemi, Sanja Avramovic, Mark Schwartz.

## Supplementary Material




